# Supplementary material for: A Rotatable Paper Device Integrating Reverse Transcription Loop-Mediated Isothermal Amplification and a Food Dye for Colorimetric Detection of Infectious Pathogens
Source: Biosensors (Basel). 2022 Jul 4;12(7):488. doi: 10.3390/bios12070488 (PMC9313173; doi:10.3390/bios12070488)
Supplement: Supplementary file 1 [file biosensors-12-00488-s001.zip › biosensors-1762928-supplementary.pdf]

biosensors

MDPI

Supplementary materials

A Rotatable Paper Device Integrating Reverse Transcription Loop-Mediated Isothermal Amplification and a Food Dye for Colorimetric Detection of Infectious Pathogens

Hanh An Nguyen, Heewon Choi and Nae Yoon Lee *

Department of BioNano Technology, Gachon University, 1342 Seongnam-daero, Sujeong-gu, Seongnam-si 13120, Gyeonggi-do, Korea; hanhannguyen96@gmail.com (H.A.N.); heewonchoi928@gmail.com (H.C.)

* Correspondence: nylee@gachon.ac.kr; Tel.: +82-31-750-8556

Materials. Copper (II) sulfate pentahydrate and carmoisine were purchased from Sigma-Aldrich. The RT-LAMP reagents, composed WarmStart RTx Reverse Transcriptase, *Bst* 2.0 WarmStart DNA Polymerase, 10× isothermal amplification buffer, dNTP mix, and 100 mM MgSO4 was obtained from New England Biolabs (Ipswich, MA, USA). For chip fabrication, Whatman filter paper grade 2 was purchased from GE Healthcare Life Sciences; Kent paper was purchased from a stationery shop; poly(methyl methacrylate) (PMMA) substrate was obtained from Goodfellow. Polydimethylsiloxane (PDMS) prepolymer (SYLGARD 184) and a curing agent were purchased from Dow Corning. AMPLIRUN® SARS-CoV-2 RNA CONTROL, AMPLIRUN® CORONAVIRUS OC43 RNA CONTROL, and AMPLIRUN® CORONAVIRUS SARS (2003) RNA CONTROL were supplied by Vircell (Granada, Spain). For gel electrophoresis, agarose powder was purchased from BioShop (Burlington, ON, Canada), Loading STAR from Dyne Bio (Seongnam, Korea), and DNA ladder (100 bp) from Takara (Shiga, Japan). To detect the target bands, Gel Doc EZ System (Bio-Rad, Hercules, CA, USA) was used.

Table S1. Primer sequences used for the detection of SARS-CoV-2, SARS-CoV, HCoV-OC43.

Target gene	Primer name	Primer sequences (5′-3′)
E gene (SARS-CoV-2)	LB	GCTGCAATATTGTTAACGTGAGTC
	F3	TCATTCGTTTCGGAAGAGA
	B3	AGGAACTCTAGAAGAATTCAGAT
	FIP	TGTAACTAGCAAGAATACCACGAAA-CAGGTACGTTAA-TAGTTAATAGCG
	BIP	GCTTCGATTGTGTGCGTACT-CGAGAGTAAACGTAAAAA-GAAGG
E gene (SARS-CoV)	LB	AACGTGAGTTTAGTAAAACCAACGG
	F3	GTTTCGGAAGAAACAGGTAC
	B3	CTCCTTCAGAAGAGTTCAGAT
	FIP	CGCAGTAAGGATGGCTAGTGTG-GCGTACTTCTTTTTCTT-GCTT
	BIP	CTTCGATTGTGTGCGTACTGC-TTAACACGCGAGTAGACGT
N2 gene (HCoV-OC43)	LF	CCCGATCGACAATGTCAGC
	F3	GCACCGATATTGACGGAGTC
	B3	ACTACGCGATCCTGCACTA
	FIP	CAGGCGGAAACCTAGTCGGAAT-GGCTGATGTCAA-TACCCCG
	BIP	TTGAAGGCTCAGGAAGGTCTGC-GAGGCTCTGCTGGATGTG
esp gene (*Enterococcus faecium*)	LB	TGATGTTGACACAACAGTTAAGGG
	F3	CCAGAACACTTATGGAACAG
	B3	GTTGGGCTTTGTGACCTG
	FIP	CGTGTCTCCGCTCTCTTCTTT-TTATTTGCAAGA-TATTGATGGTG
	BIP	ATCGGGAAACCTGAATTAGAAGAAG-AACTCGTGGATGAA-TACTTTC

Table S2. Comparison between the rotatable paper device and previous techniques.

Target	LOD	Time	Naked-eye detection	Ref
HCoV-OC43	10^3 copies/μL	70 min	Yes	Current study
	2 × 10^2 copies/μL	96 min	No	[1]
SARS-CoV-2	10^3 copies/μL	70 min	Yes	Current study
	10^2 copies/μL	30 min	No	[2]
SARS-CoV	10^3 copies/μL	70 min	Yes	Current study
	10 copies /μL	145 min	No	[3]

1. Vijgen, L.; Keyaerts, E.; Moës, E.; Maes, P.; Duson, G.; Ranst, M.V. Development of One-Step, Real-Time, Quantitative Reverse Transcriptase PCR Assays for Absolute Quantitation of Human Coronaviruses OC43 and 229E. *J. Clin. Microbiol.* **2005**, *43*, 5452–5456.

2. Yoon, Taehwi.; Shin, J.; Choi, H-J.; Park, K.S. Split T7 promoter-based isothermal transcription amplification for one-step fluorescence detection of SARS-CoV-2 and emerging variants. *Biosens. Bioelectron.* **2022**, *208*, 114221.

3. Escutenaire, S.; Mohamed, N.; Isaksson, M.; Thorén, P.; Klingeborn, B.; Belák, S.; Berg, M.; Blomberg, J. SYBR Green real-time reverse transcription-polymerase chain reaction assay for the generic detection of coronaviruses. *Arch. Virol.* **2007**, *152*, 41–58.

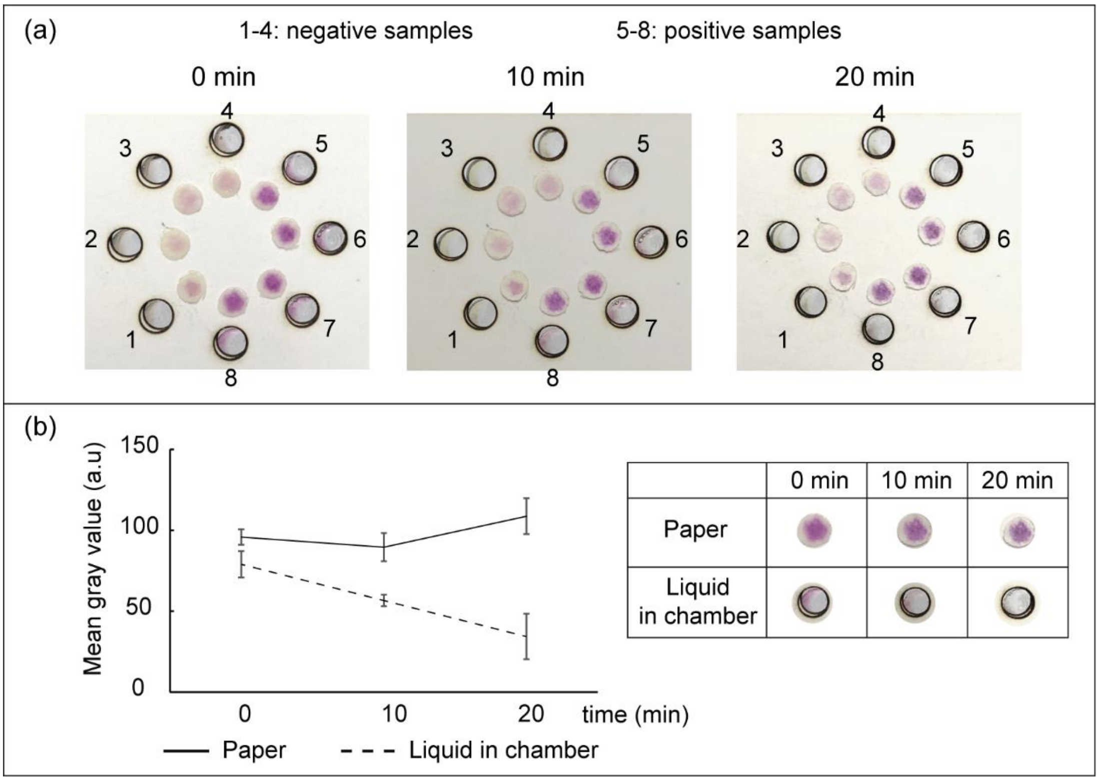

Figure S1. Influence of time on the stability of color of carmoisine. After carmoisine-based detection was performed on the paper-based device, 2 μL of RT-LAMP amplicons was loaded onto the paper discs on the paper-based device. The paper-based device was left at room temperature for 0 min, 10 min, and 20 min to compare the color change on the paper and the liquid in the reaction chambers. (**a**) Results showing the color change on the paper and the liquid after 0 min, 10 min, and 20 min. (**b**) Mean gray values obtained from ImageJ. All the experiments were repeated three times.

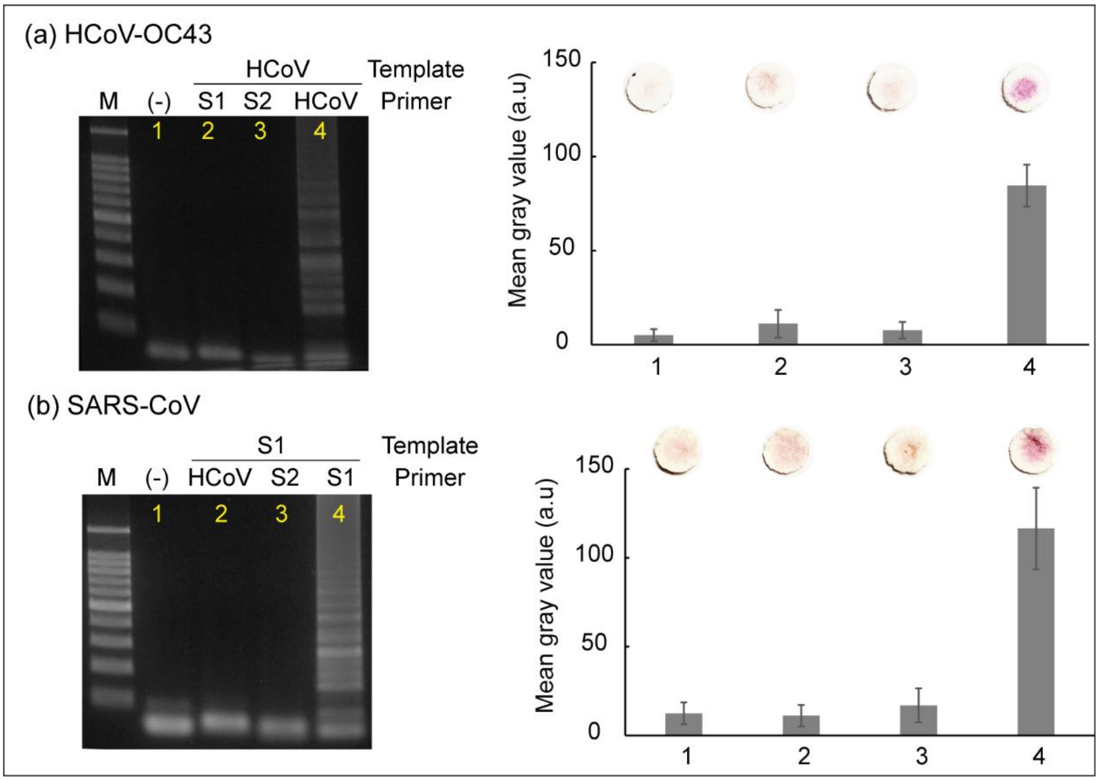

Figure S2. The results of the specificity test for (**a**) HCoV-OC43 detection and (**b**) SARS-CoV detection. All the experiments were repeated three times.

As Fig. S2 shows, primer set for HCoV-OC43 amplification was used to detect SARS-CoV, SARS-CoV-2, and HCoV-OC3. From lane 2 to 4, HCoV-OC43 primers were added into samples containing SARS-CoV, SARS-CoV-2, and HCoV-OC43 RNA, respectively. Lane 1 can be consider as negative control because RT-LAMP was performed without RNA template. As results, only sample containing HCoV-OC43 was successfully amplified. Similarly, only SARS-CoV was successfully amplified using SARS-CoV primer.

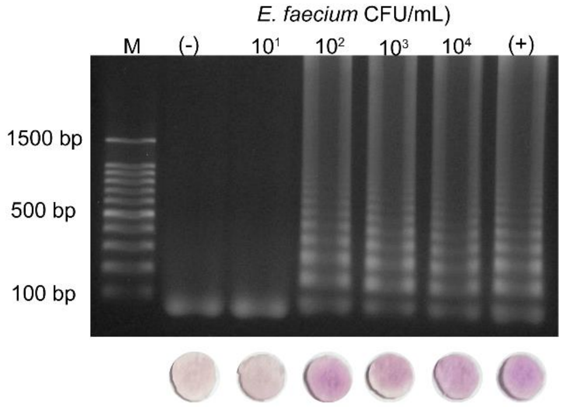

Figure S3. Results showing the sensitivity of *E. faecium* detection mediated by carmoisine.

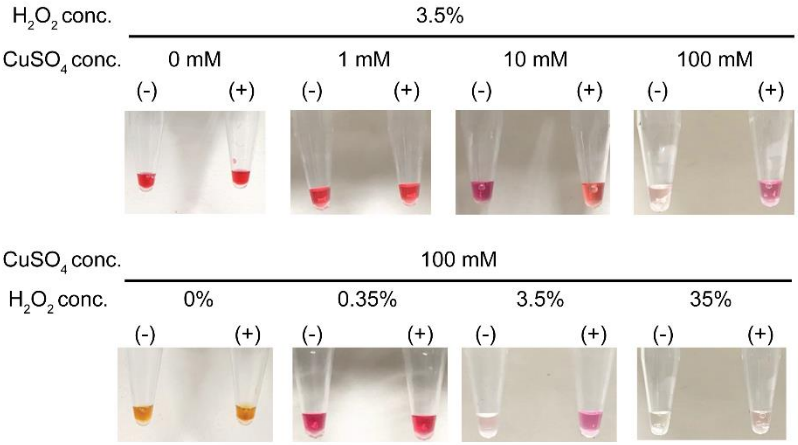

Figure S4. Optimization of carmoisine-based detection.
